# Detection of Honey Adulteration Using Thermorheological and Spectroscopic Analyses: Independent Evaluation With Linear Latent‐Variable and Gradient Boosting Models

**DOI:** 10.1111/1750-3841.71228

**Published:** 2026-06-26

**Authors:** Bilge Basturk Berk, Berkay Berk, Cagri Cavdaroglu, Neslihan Bozdogan, Sebnem Tavman, Seher Kumcuoglu, Sevcan Unluturk

**Affiliations:** ^1^ Department of Food Engineering Faculty of Engineering Ege University İzmir Türkiye; ^2^ Department of Food Engineering Faculty of Engineering Izmir Institute of Technology İzmir Türkiye

**Keywords:** adulteration, artificial intelligence, fluorescence, honey, thermorheology, UV‐Vis

## Abstract

Honey adulteration is a prevalent economic fraud that demands robust and reliable detection methods. In this study, a proof‐of‐concept was developed on a comparative study of oscillatory thermorheology (20°C–80°C) and spectroscopic techniques, including UV‐Vis and fluorescence spectroscopy, for the detection of adulteration with glucose, invert sugar, and maltose syrups. A total of 86 samples were analyzed, comprising authentic blossom and honeydew honeys, as well as samples adulterated at concentrations ranging from 5% to 50%. Thermorheological and spectroscopic datasets were analyzed independently to assess their individual discriminative power. For each analytical approach, classification performance was evaluated using linear latent‐variable models (PLS‐DA, OPLS‐DA) and gradient‐boosting machine learning techniques (LightGBM, XGBoost), enabling a systematic comparison between classical chemometric and nonlinear machine‐learning methods. The results revealed clear performance differences between the analytical approaches and modeling strategies. The results demonstrated that while spectroscopic models achieved high sensitivity (>0.96), they frequently failed to correctly identify authentic samples, resulting in notably low specificity (0.17‐0.42). In contrast, thermorheological parameters more effectively captured the structural and physicochemical alterations induced by the addition of glucose, invert sugar, and maltose syrups. Notably, loss modulus data modeled using the LightGBM algorithm achieved the most balanced classification performance, reaching 0.75 specificity and 92.67% external validation accuracy while maintaining high sensitivity. These findings demonstrate that thermorheological analysis, particularly when evaluated using advanced non‐linear gradient boosting models, offers superior discriminative capability for honey authentication compared to spectroscopic analysis alone.

## 1 Introduction

Honey is a naturally sweet food composed primarily of fructose and glucose in an aqueous matrix. Owing to its nutritional value and corresponding economic worth, honey is among the food products most frequently targeted by fraud, particularly adulteration. In this context, adulteration refers to the addition of cheaper food materials to increase the apparent amount of a valuable food product, a practice commonly described as economically motivated adulteration (EMA) (Berk et al. 2022; Everstine et al. 2013; Momtaz et al. 2023). Addition of any material to honey is explicitly prohibited under Codex Alimentarius (Codex Alimentarius Commission 2001). Honey adulteration is often done using lower‐cost corn, rice, and invert sugar syrups. The corn and rice syrups are starch‐based sweeteners, while the invert sugar syrup is produced from sucrose. The sugar profile, water content, density, and viscosity of honey adulterated with these sugar substitutes could be easily adjusted so that consumers cannot detect the adulteration. For instance, the moisture content can be adjusted (typically to around 80–83 °Bx) through evaporation or dilution of sugar syrups to closely replicate the density of genuine honey. In addition, viscosity can be modified by blending low‐viscosity inverted sugars with thicker syrups, such as rice or corn syrup, to achieve a balance of dextrins and oligosaccharides similar to that of natural honey. Since these basic physical properties can be easily masked or altered, more advanced analytical methods are necessary for accurate authentication.

In the last couple of decades, various analytical methods were developed and studied for the determination of honey adulteration, including isotope ratio mass spectrometry (Çinar et al. 2014), three‐dimensional fluorescence spectroscopy (Q. Chen et al. 2014), high‐performance liquid chromatography with a diode array detector (Xue et al. 2013), gas chromatography with mass spectrometry (Ruiz‐Matute et al. 2010), time domain nuclear magnetic resonance (NMR) (Berk et al. 2022), and nuclear magnetic resonance spectroscopy (Bertelli et al. 2010). While these advanced techniques offer high sensitivity and detailed molecular profiling, their routine industrial application is often hindered by high equipment and maintenance costs, the requirement for highly skilled operators, and time‐consuming sample preparation procedures.

In the study of Huang et al. (2020), natural and syrup‐adulterated honey was identified by integrating near‐infrared (NIR) and mid‐infrared (MIR) spectral data and building a support vector machine (SVM) classifier with low‐ and intermediate‐level data fusion, where SVM parameters (C and γ) were optimized using grid search, genetic algorithms, and particle swarm optimization. Compared to single‐spectrum models and low‐level fusion, intermediate‐level fusion improved performance and achieved 100% accuracy, specificity, and sensitivity in distinguishing natural honey from syrup‐adulterated honey. In another study by Fakhlaei et al. (2025), honey adulteration was detected and quantified using attenuated total reflectance accessory equipped Fourier transformed infrared spectroscopy (FTIR‐ATR) spectra (3600‐400 cm^−1^) coupled with partial least squares (PLS) regression on bee honey and stingless bee honey adulterated with acids (acetic acid, citric acid, and tamarind extract; 1%–7%) and sugars (liquid corn syrup, cane sugar, palm sugar, and inverted sugar; 1%–3%), supported by physicochemical and sugar‐profile measurements (total soluble solid content, moisture, pH, free acidity, glucose, fructose, and sucrose). Compared to conventional physicochemical testing alone, the FTIR‐ATR combined with the PLS approach achieved strong predictive performance (R^2^ up to 0.99 depending on the adulterant) and correctly classified all 78 samples in validation, indicating a fast, reliable, non‐destructive method for multi‐adulterant honey authentication.

Dimakopoulou‐Papazoglou et al. (2023) identified honey adulteration with sugar syrups and colorants using UV‐Visible (UV‐Vis) spectroscopy (220‐550 nm) combined with chemometrics on 209 samples comprising 151 commercial Mediterranean honeys (thyme, pine, and polyfloral) from Greece, Malta, Spain, Tunisia, and Türkiye and 58 adulterated Greek thyme honeys. Chemometric models achieved high accuracy and specificity (with most models exceeding 90% prediction accuracy), indicating that UV‐Vis spectroscopy combined with multivariate analysis is a rapid, inexpensive, and non‐destructive approach for honey authenticity assessment.

Fluorescence spectroscopy is another technique used for detecting honey adulteration, where emission spectra (385‐800 nm; λ_ex_ = 370 nm) and, in particular, the frequency‐doubled peak (FDP) intensity at 740 nm (together with the emission apex wavelength) are monitored to screen for sugar syrup addition. In this approach, FDP intensity decreases significantly, and the emission apex shows a dose‐dependent blueshift, enabling detection of ≥10% syrup and yielding approximately 75% accuracy in a validation set of 20 adulterated samples (Yan et al. 2022).

The study of predicting honey adulteration through dynamic rheological testing has not received as much attention. However, there are studies in the literature that provide valuable information about the viscoelastic properties of honey without damaging its structural elements through small‐deformation oscillatory rheology. For example, Yilmaz et al. (2014) conducted a study to analyze the rheological behavior of honey samples adulterated with sucrose and fructose syrups. Their results demonstrated a decrease in viscosity as a result of the added adulterants, establishing a clear link between rheological properties and sugar composition. Moreover, multivariate statistical data analysis has been employed to distinguish adulterated honey samples and classify them based on their sugar composition and rheological parameters. In studies on honey adulteration, researchers examine how added substances change the composition of honey or cause irregularities in its components. This process results in the generation of large datasets. To analyze the complex structure of data and compare similarities or differences in a large data set, multivariate statistical analysis is needed. There are different studies in the literature on this subject. For instance, Amiry et al. (2017) applied multivariate analysis to the rheological and physicochemical properties of honey using principal component analysis (PCA) followed by a linear discriminant analysis (LDA) to classify the samples, based on type and concentrations of adulterants (date and invert syrups). This study also demonstrated the acceptability of PCA and LDA in distinguishing complex sugar adulteration in honey. Moreover, rheological properties were able to determine the concentration of adulteration as well as the adulteration with complex sugars. In another study conducted by Oroian et al. (2018), rheology was used in the detection of honey adulteration. Different rheological variables were used in multivariate statistical analysis and found that even a 5% addition of sugar syrup can be detected by PCA.

To strengthen the predictive properties of the commonly used methods, there are continuous developments in techniques. Enhancing the models with machine learning (ML) provides faster and more accurate results. Linear and non‐linear chemometric techniques can be advantageous or disadvantageous depending on data modality, pre‐processing, and adulterant‐driven signal complexity. In a study conducted by Yeole et al. (2024), hyperspectral imaging results were processed using the Light Gradient Boosting Method (LightGBM) and eXtreme Gradient Boosting (XGBoost) to evaluate the honey adulteration determination. Using the boosting technique, they achieved 90.37% and 91.97% accuracy for LightGBM and XGBoost, respectively.

NMR and IR‐based methods can detect honey adulteration with high accuracy, but their routine deployment is often constrained by instrument cost and maintenance requirements. In contrast, UV‐Vis and fluorescence spectroscopy are widely available in lower‐budget laboratories, enable rapid measurements, and typically impose lower operational and analytical costs. Accordingly, the proposed method is positioned as a practical, cost‐effective alternative for routine monitoring in resource‐limited settings rather than a replacement for high‐cost analytical platforms. Accordingly, the proposed method is positioned as a low‐cost alternative similar to UV‐Vis and fluorescence‐based screening for routine honey authenticity monitoring in resource‐limited settings, rather than as a substitute for high‐end platforms such as NMR or IR. To the authors’ knowledge, this is the first study proposing the application of new generation data analysis methods to detect honey adulteration using rheology. Thermorheological analysis was assessed as an independent approach for screening honey authenticity.

This proof‐of‐concept study focuses on the independent detection of honey adulteration using thermorheological and spectroscopic analyses. Oscillatory temperature sweep tests, UV–Visible spectroscopy, and fluorescence spectroscopy were applied to authentic and adulterated honey samples prepared with three adulterants: glucose syrup (S1), invert sugar syrup (S2), and maltose syrup (S3). Rheological and spectral datasets were analyzed separately to develop predictive models for classifying authentic and adulterated samples.

Both linear methods, including partial least squares discriminant analysis (PLS‐DA) and orthogonal partial least squares discriminant analysis (OPLS‐DA), and non‐linear gradient boosting methods, including LightGBM and XGBoost, were implemented to systematically compare the discriminative performance of thermorheological and spectroscopic data and to evaluate the relative effectiveness of linear and non‐linear modeling strategies in honey adulteration detection. Unlike previous studies primarily focused on conventional chemometric approaches, this work provides a comprehensive comparison between latent‐variable classifiers and advanced gradient boosting models, while highlighting the potential of thermorheological data within high‐performance machine learning workflows.

## 2 Materials and Methods

### 2.1 Materials and Sample Preparation

One authentic blossom honey sample was from the General Directorate of Agricultural Enterprises (TIGEM Vakfı) (A01) and the other one was from Ege University, Faculty of Agriculture (A03). The other two authentic honeydew honey samples (pine—A02) and (chestnut—A04) were sourced from certified local producers in the Marmaris and Kastamonu regions of Türkiye, respectively. The blossom honey samples were obtained from the İzmir and Dalaman regions of Türkiye. All samples were harvested in 2022. Their electrical conductivity, water activity, and total soluble solids ranged from 2.55 to 8.71 × 10^−4^ S/cm, 0.570 to 0.602, and 81.9 to 83.1 °Bx, respectively. Besides, commercial authentic samples (five different blossom honey and five different honeydew honey samples) were also purchased from the local market in İzmir, Turkey. Their authenticity was checked prior to adulteration based on the method as described in Section 2.2.1. In addition, before adulteration, the total soluble solid (TSS) content of the sugar syrups was adjusted to 82 °Brix, which was equal to the TSS content of honey. To prepare adulterated samples, these four authentic honeys (A01, A02, A03, and A04) were mixed with different adulterants such as glucose syrup (S1) (Tito, Smart Kimya, Türkiye), invert sugar syrup (S2) (Konya Seker, Türkiye), and maltose syrup (S3) (Tito, Smart Kimya, Türkiye) separately at 5, 10%, 20%, 30%, 40%, and 50% concentrations. A total of 86 samples were used in this study; 72 of them were adulterated, and 14 of them were authentic samples.

### 2.2 Methods

#### 2.2.1 Material Characterization

Honey samples were analyzed to check their authenticity by isotope ratio mass spectrometry (IR‐MS) with the internal method (modified from AOAC 998.12) of Ege University Research and Application Center of Drug Development and Pharmacokinetics (ARGEFAR, İzmir, Turkey). All the honey samples were found to be authentic by their C4 sugar ratio being less than 2%.

The sugar profile of the adulterant syrups (S1, S2, and S3) were analyzed by the method of Cavdaroglu and Ozen (2022) with the system consisting of HPLC having a refractive index detector (Agilent 1200, Santa Clara, CA, USA) and an Aminex 87H column (300 × 7.8 mm, 9 µm, Bio‐Rad Laboratories, Hercules, CA, USA) in duplicated measurements. Concentration of the sugars was determined from standard curves. The composition of the sugar syrups is given in Table 1.

**TABLE 1:** Sugar composition of the sugar syrups.

Syrup	Total soluble solid (°Bx)	Fructose (g kg^−1^)	Glucose (g kg^−1^)	Sucrose (g kg^−1^)	Maltose (g kg^−1^)	DP_n_^†^ (g kg^−1^)
S1	81.80 ± 0.30	6.50 ± 0.14	159.70 ± 5.66	—	165.10 ± 0.14	450.20 ± 2.16
S2	82.70 ± 0.53	307.55 ± 2.62	290.85 ± 1.34	259.60 ± 11.45	—	—
S3	81.60 ± 0.23	5.45 ± 0.49	20.5 ± 0.71	—	402.30 ± 1.25	370.30 ± 9.66

S1: invert syrup, S2: glucose syrup, S3: maltose syrup, DP_n_:sugar consisting of the remaining sugar.

#### 2.2.2 Rheological Measurements

The dynamic rheological properties of both authentic and adulterated samples were measured using a hybrid rheometer (DHR‐3, TA Instruments, New Castle, DE, USA) equipped with a Peltier plate heating system and a cone‐and‐plate geometry with a 40 mm diameter and a 2° cone angle. The strain sweep test was conducted to determine the linear viscoelastic region (LVR) in the range of 0.001‐100% at the frequency of 1 Hz (25°C), then, an oscillatory temperature sweep test was performed from 20°C to 80°C at the scanning rate of 3°C at 1% strain (within LVR) and 1 Hz oscillation frequency. Storage modulus (G′) and loss modulus (G′′) data were obtained from rheological measurements and loss tangent (tan δ), which is the ratio between the viscous and elastic properties of a material, was calculated from the storage and loss modulus. Each measurement was repeated at least three times.

Prior to measurement, all samples were kept in a 50°C water bath for 1 h to dissolve honey crystals, if any, to prevent their interference during the shearing process (Berk et al. 2022).

#### 2.2.3 Differential Scanning Calorimetry

Differential scanning calorimetry (DSC) analysis was conducted on authentic samples and the sugar syrups using a calorimeter (DSC Q10, TA Instruments, New Castle, DE, USA). Samples weighed about 5 mg and were heated from 0°C to 100°C at the heating rate of 10°C/min with a 50 mL/min N_2_ gas purge rate. From the resulting thermograms, the onset temperature (T_onset_) and the calorimetric enthalpy (Δ_cal_H) of the thermal transitions were determined.

#### 2.2.4 UV‐Visible Spectroscopy

UV‐Vis spectra of all samples were recorded in the 280–900 nm range using a UV‐Vis spectrophotometer (Thermo Scientific Varioskan, Fisher Scientific, Vantaa, Finland). Measurements were done by direct loading of 200 µL of samples to the transparent 96‐well flat bottom polystyrene plate (Isolab, Wertheim, Germany). The spectrum of each sample was collected twice, and they were averaged.

#### 2.2.5 Fluorescence Spectroscopy

Fluorescence spectra of all samples were obtained in 320–550 nm with 1 nm intervals by using a fluorescence spectrophotometer (Thermo Scientific Varioskan, Fisher Scientific, Vantaa, Finland). Samples were excited at a 300 nm wavelength. The slit width was adjusted to 12 nm. Measurements were done by direct loading of 200 µL of samples to the black 96‐well flat bottom polystyrene plate (Isolab, Wertheim, Germany). The spectrum of each sample was collected twice, and they were averaged.

#### 2.2.6 Statistical Analysis

Rheological data and UV‐Vis and fluorescence spectral data of authentic and adulterated honey samples were analyzed separately using linear latent‐variable methods (PLS‐DA and OPLS‐DA) and non‐linear gradient boosting algorithms (LightGBM and XGBoost) to differentiate between pure and adulterated samples. Observations were divided into calibration and external validation subgroups using a stratified random sampling method based on adulteration percentiles, and random sampling was performed within these subgroups (Särndal et al. 1992). Two‐thirds of the collected observations were utilized for calibration, and the remaining observations were used for external validation. For each Monte Carlo repetition, spectral transformations were applied independently to calibration and hold‐out subsets after partitioning. Preprocessing selection was based solely on calibration‐set cross‐validation performance (Q^2^/AUC), and the hold‐out set remained blind throughout model development and preprocessing optimization.

In addition to raw data, the data were subjected to various preprocessing transformations, including the first derivative (FD), second derivative (SD), third derivative (TD), Savitzky‐Golay smoothing (SG), standard normal variate (SNV), and multiplicative scatter correction (MSC). Additionally, combinations of derivatives with scatter correction techniques were applied (FD + SNV, FD + MSC, SD + SNV, SD + MSC, TD + SNV, and TD + MSC).

PLS‐DA and OPLS‐DA analyses were conducted using the “ropls package” (Version 3.12) in the R programming language (Thévenot et al. 2015). The N4 and R1 criteria were used to guide latent‐variable selection in the PLS and OPLS models. Any component was removed when its increment $R_{Y}^{2}$ was below 0.01 or its $Q_{Y}^{2}$ was negative; components not meeting these removal thresholds were retained. Together with tracking R^2^ in the calibration and external validation sets, the use of the N4 and R1 rules supported parsimonious modeling and reduced the risk of overfitting.

The LightGBM algorithm was employed in the R programming language using the “lightgbm” package (Shi et al. 2023). The objective function was set to binary classification, and the learning rate was set to 0.1. The maximum number of leaves per decision tree was set to 31. The model was trained for 100 boosting iterations using the prepared training dataset. The XGBoost algorithm was also utilized to develop a binary classification model using the “xgboost” package (T. Chen et al. 2022). The objective function was defined as logistic regression, and the maximum tree depth was limited to 2. A learning rate of 0.05 was chosen, while subsampling was applied to both the training instances (60%) and the features (60%) used at each tree‐building iteration. The model was trained using 100 boosting rounds on the prepared training dataset, with the verbosity level set to zero. Model performance was assessed using the area under the receiver operating characteristic curve (AUC) as the evaluation metric. Early stopping was implemented with a threshold of 10 consecutive rounds without improvement in the validation metric. Analytical parameters for Light GBM and XGBoost were kept at the default values of the respective R packages except where specifically noted.

To ensure model robustness and prevent overfitting, the optimal number of latent variables and hyperparameter configurations were determined using 7‐fold internal cross‐validation within the calibration set. For each unique combination of data source, preprocessing method, and chemometric model, this entire procedure was repeated three times with new random sampling for each iteration. All performance metrics, including calibration and external validation results, were expressed as the mean and standard deviation to verify the statistical stability and reproducibility of the classification outcomes. An analysis of variance (ANOVA) test and Tukey's post‐hoc test were applied at the 95% confidence level to these results. The selection of preprocessing transformations followed a systematic pipeline tailored to the specific physical characteristics of each data source. For spectroscopic data, techniques such as SNV and derivatives were employed to neutralize baseline shifts and scattering effects, while thermorheological data were scaled to ensure numerical stability across the temperature gradient. The robustness of this approach was confirmed by the minimal discrepancy between calibration (correct classification rate for calibration—CCR_c_) and external validation (correct classification rate for validation—CCR_v_) performance, indicating that the selected transformations enhanced the diagnostic signal without compromising model generalization.

The adequacy of the classification models was primarily evaluated using the CCR_v_, which served as the key metric for predictive success. To ensure model robustness and safeguard against overfitting, the discrepancy between calibration $R_{Y}^{2}$ and validation $Q_{Y}^{2}$ performance was rigorously monitored, while sensitivity and specificity were utilized as secondary indicators to verify the diagnostic balance and reliability of the outcomes.

(1) $$\mathrm{Sensitivity}\;=\frac{\mathrm{True}\;\mathrm{Positive}}{\mathrm{True}\;\mathrm{Positive}\;+\;\mathrm{False}\;\mathrm{Negative}}\;$$

(2) $$\mathrm{Specificity}\;=\frac{\mathrm{True}\;\mathrm{Negative}}{\mathrm{True}\;\mathrm{Negative}\;+\;\mathrm{False}\;\mathrm{Positive}}\;$$

(3) $$\mathrm{Correct}\;\mathrm{Classification}\;\mathrm{Rate}\;=\frac{\mathrm{True}\;\mathrm{Positive}\;+\;\mathrm{True}\;\mathrm{Negative}}{\mathrm{Total}\;\mathrm{Number}\;\mathrm{of}\;\mathrm{Samples}}\;$$

The specificity, sensitivity, and correct classification rate for calibration (cross‐validation) and external validation were calculated as shown above Equations (1), (2), (3), where the positive class corresponds to adulterated honey samples and the negative class corresponds to authentic (non‐adulterated) honey samples; thus, true negatives are correctly identified authentic samples, and true positives are correctly identified adulterated samples.

## 3 Results and Discussion

### 3.1 Rheological Properties

**FIGURE 1:**
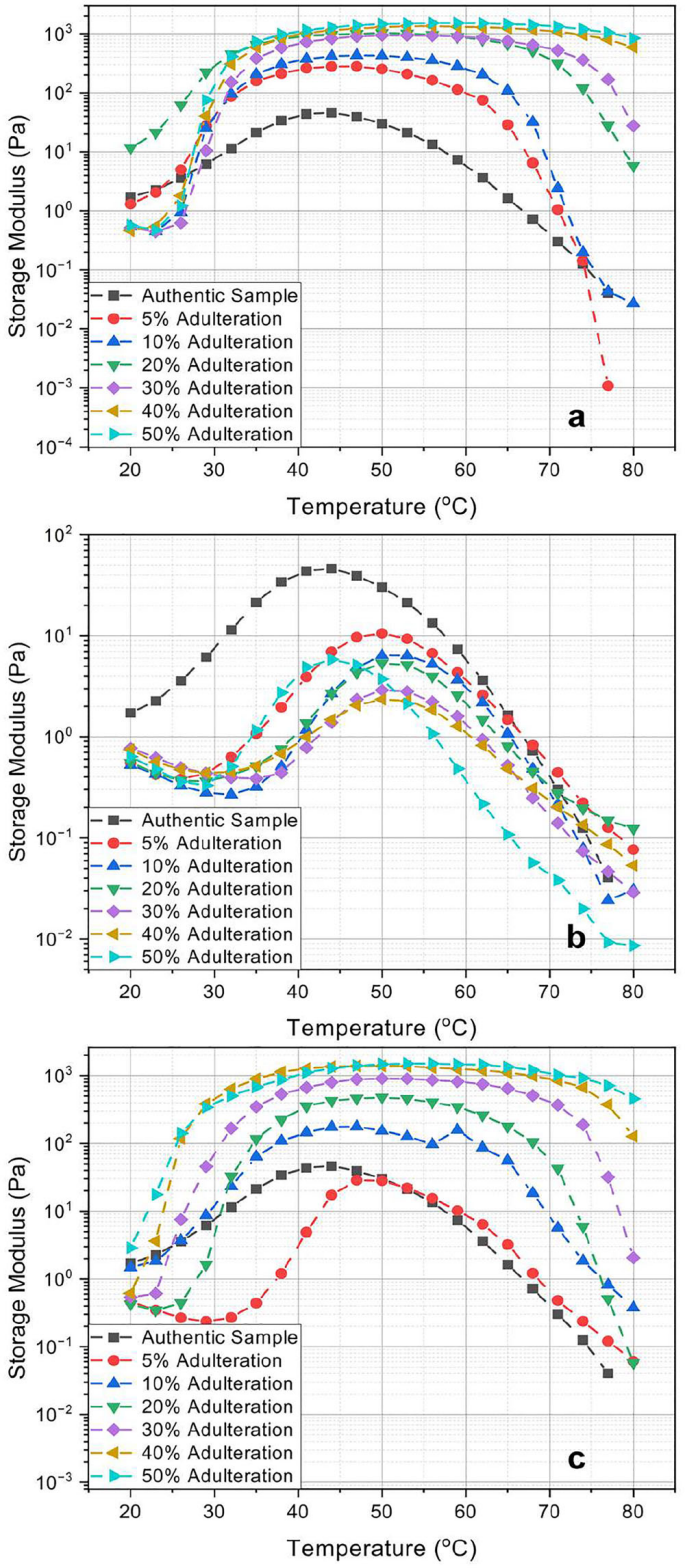
Storage moduli of a representative authentic honey (Sample A04) and its corresponding adulterated mixtures with (a) glucose, (b) invert sugar, and (c) maltose syrups. Data for all other sample matrices are available in Supplementary File A.

**FIGURE 2:**
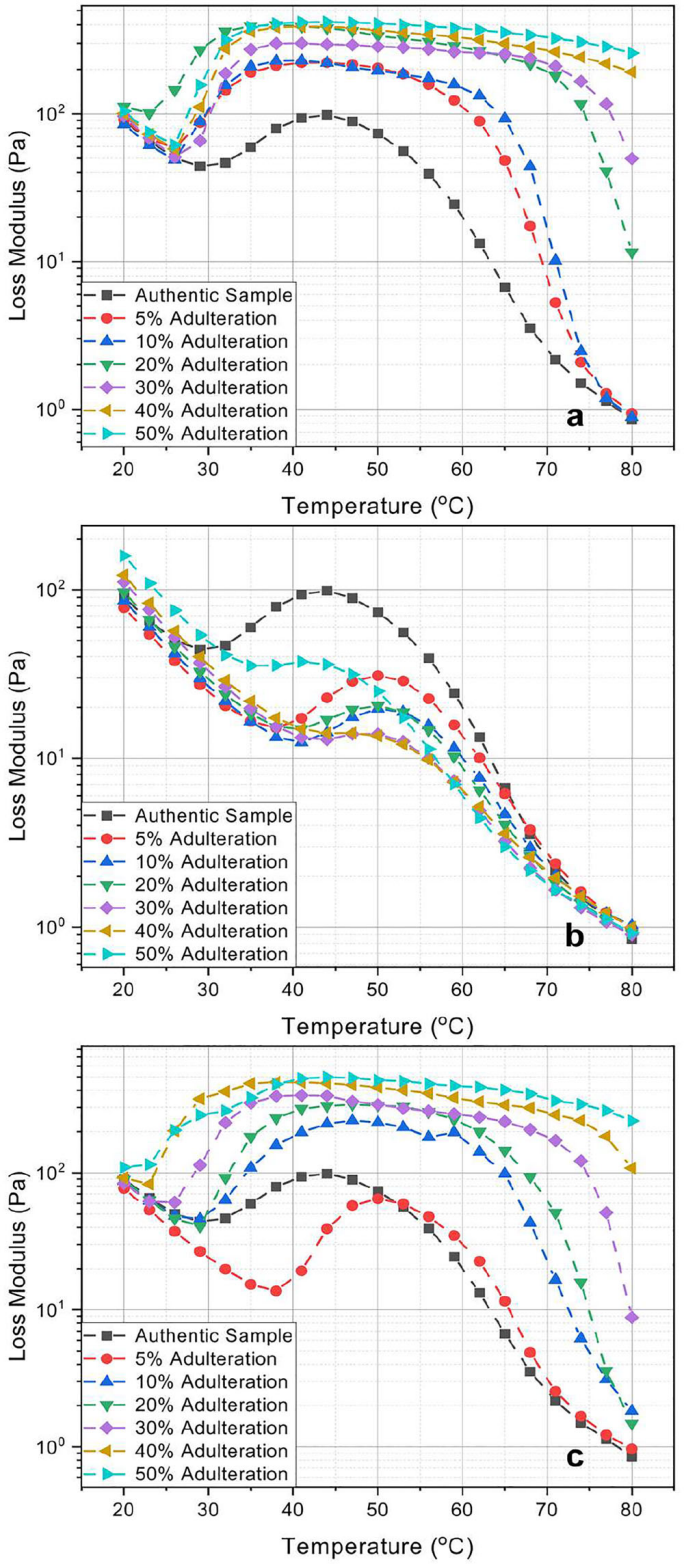
Loss moduli of a representative authentic honey (Sample A04) and its corresponding adulterated mixtures with (a) glucose, (b) invert sugar, and (c) maltose (c) syrups. Data for all other sample matrices are available in Supplementary File A.

The magnitude of storage (G') moduli, magnitude of loss (G“) moduli, and tan δ (tangent of the phase angle), a ratio between viscous and elastic properties, of the authentic and adulterated samples were evaluated. For clarity of visualization, the rheological profiles of a single representative authentic sample (Sample A04) and its corresponding adulteration gradients are presented in Figures 1 and 2. Attempting to plot all 86 samples on a single axis obscures the distinct temperature‐dependent structural transitions; however, the trends discussed below were consistent across the other authentic bases. The comprehensive graphical dataset for all samples is provided in Supplementary File A. In addition, crossover points for G' and G”, which indicate the transition between viscous and elastic behavior of honey, were shown in Table S1 (Supplementary File B). The samples exhibited different behaviors with temperature depending on these factors. The magnitudes of the storage and loss moduli, as well as the crossover points, were significantly influenced by the syrup's characteristics and the adulteration percentage.

In the authentic samples, the magnitude of G'' was larger than G', indicating that these samples exhibited more viscous behavior. As the temperature increased, both the loss and storage moduli decreased in all authentic samples. Li et al. (2013) reported similar findings in crystallized honey samples, where changes in G' with increasing temperature were divided into three distinct regions. The first stage, known as the softening region, occurs when molecules gain enough thermal energy to begin moving or revolving, though this was not observed in our study. The second stage, referred to as the crystalline plateau region, was observed when G' lies between 20 and 30°C, extending up to the sample's melting point. The final stage, the melting region, is reached when temperatures exceed the crystalline plateau, leading to a significant decrease in G' as the crystals dissolve.

Adulteration with glucose syrup (S1) resulted in an increase in both the storage and loss moduli, leading to the occurrence of crossover points throughout the temperature scan, with higher adulteration concentrations further increasing the magnitudes of both moduli. In the A02 and A03 samples, temperature scans revealed that the storage and loss moduli for samples with 5% to 30% adulteration crossed each other at two distinct temperatures, whereas samples with 40% and 50% adulteration exhibited only one crossover point. Similarly, in the A04 sample, two crossover points were observed for samples with 5% to 20% adulteration, while those with 30%, 40%, and 50% adulteration showed only one. Conversely, in the A01 sample, the 30%, 40%, and 50% adulteration levels exhibited a single crossover point, whereas no crossover points were observed in samples with 5%, 10%, and 20% adulteration. All samples displayed viscous‐like behavior in lower temperatures (<25°C). With the increase in temperatures, samples with adulterants started to display solid‐like behavior after 25°C–40°C. As given in Table 1, the glucose syrup (S1) has the highest amount of sugar polymer, and the starch is the main substrate for the glucose syrup production (Amaral‐Fonseca et al. 2020). The elastic behavior observed at higher temperatures could be the reason for the gelatinization of starch present in the adulterant. As the amount of adulterant increases, the magnitude of the storage modulus increases and remains constant. Dynamic rheological analysis revealed that glucose syrup exhibits solid‐like behavior with increasing temperature, while honey remains viscous throughout the temperature range. This behavior change suggests that the presence of at least 5% glucose syrup in the A02, A03, and A04 samples can be detected within the temperature range of 20°C–40°C. Conversely, in the A01 sample, the detection of glucose syrup is limited to concentrations of 30% or higher, which is detectable around 40°C.

In the samples adulterated with inverted sugar syrup (S2), both the storage and loss modulus magnitudes were decreased as the adulteration concentration increased. For all samples, the storage modulus values were lower than the loss modulus values, and there was no crossover point observed, suggesting adulterated samples displayed a viscous nature similar to what is characteristic of liquid‐like macromolecule solutions. However, from 30°C to 50°C, tan δ decreased. Around 50°C, the ratio of the loss modulus to storage modulus reached the lowest, meaning relatively less liquid‐like behavior. Despite this, no crossover point was observed, and after 50°C the ratio of the loss modulus to storage modulus started to increase. The composition of invert sugar syrup was given in Table 1, and it is composed of nearly equal amounts of glucose, fructose, and sucrose. The addition of sucrose to the matrix alters the molecular mobility of the sucrose due to Van der Waals forces (Walther et al. 2003). Similar findings were stated by Yilmaz et al. (2014) with the natural honey adulterated with saccharose and fructose syrup. Dynamic rheological characterization of adulterated samples revealed that all samples displayed liquid‐like behavior with no crossover point. In addition, increasing the adulteration amount led to a decrease in the magnitudes of storage and loss modulus.

Maltose syrup adulteration has a similar trend to glucose syrup adulteration due to its identical origin, but it differs due to its composition. Compared to glucose syrup, it has less monomeric sugar and is mostly composed of maltose rather than the sugar polymer. The solubility of maltose is lower than that of glucose and fructose, resulting in less interaction with the matrix than the glucose syrup adulteration (Berk et al. 2022). At the beginning of the heating process, all samples exhibited similar rheological properties. As the heating process continues, the gelatinization of the sugar polymers takes place and results in elastic domination over viscous modulus. The addition of maltose syrup (S3) caused an increase in both storage and loss moduli, resulting in the appearance of crossover points during the temperature scan. Higher levels of adulteration further amplified the magnitudes of both moduli. The emergence of crossover points was found to be influenced by both the level of adulteration and the origin of the authentic sample. In the A02 sample, temperature scans indicated the presence of a crossover point in all adulterated samples. Similarly, in the A04 sample, a crossover point was observed in samples with 20% to 50% adulteration, while in the A01 and A03 samples, this point was evident at adulteration levels of 30%, 40%, and 50%. All samples exhibited viscous behavior at lower temperatures (<25°C), but as the temperature increased, samples containing adulterant began to demonstrate solid‐like behavior. In the lower adulteration amounts, less solubility of maltose and a smaller amount of sugar polymer in the matrix did not restrict the molecular mobility around the gelatinization temperature, so the samples exhibited viscous behavior during shearing. However, increasing maltose content led to the observation of crossover points, indicating that solid‐like behavior became more dominant, which can be attributed to the maltose‐water and maltose‐maltose hydrogen bonding (Li et al. 2013). With increasing amounts of maltose, both the storage modulus and loss modulus magnitude increased. However, adulterated samples with maltose syrup displayed solid‐like behavior as the temperature increased, whereas honey maintained its viscous nature across the entire temperature range.

### 3.2 Differential Scanning Calorimetry

From the thermograms of authentic honey samples and the sugar syrups, transitions were determined, and their onset temperature and the enthalpy values were given in Table 2.

**TABLE 2:** Transition temperature (T_onset_) and enthalpy (Δ_cal_H) of honeys and syrups during heating.

Sample	Transition 1		Transition 2	
	T_onset_ (°C)	Δ_cal_H_1_ (J/g)	T_onset_ (°C)	Δ_cal_H_2_ (J/g)
A01	48.26	2.29	–	–
A02	61.13	0.60	–	–
A03	57.02	2.50	–	–
A04	41.91	3.128	–	–
S1	44.86	0.19	73.94	0.18
S2	58.29	0.04	–	–
S3	47.81	0.56	82.04	0.10

A01: authentic blossom honey, A02: authentic pine honey, A03: authentic blossom honey, A04: authentic chestnut honey, S1: invert syrup, S2: glucose syrup, S3: maltose syrup.

The transition temperature (T_onset_) and enthalpy (Δ_cal_H) of authentic honey samples and syrups are given in Table 2. Different numbers of thermal phenomena were detected in the thermoanalytical curves of authentic honey samples and syrups. In the authentic honey samples and the syrup S2, one thermal phenomenon was observed, while in the S1 and S3 syrups, two thermal phenomena were observed within the temperature range of interest. Honey and the syrup samples consist mainly of fructose, glucose, maltose, sucrose, and water, whose melting points are higher than 100°C (Cardona et al. 2018; Miśkiewicz et al. 2020). Thus, these thermal phenomena can be attributed to water and volatile compound vaporization, protein denaturation, and possibly the beginning of the melting of sugars (Cardona et al. 2018).

### 3.3 UV‐Visible Spectroscopy

The color of honey serves as an indicator of the presence of pigments and is influenced by factors such as its botanical origin, the composition of the nectar, acquisition processes, temperature, and storage duration. The color spectrum of honey ranges from nearly colorless white to dark red, including shades of yellow and amber (Becerril‐Sánchez et al. 2021). Dark brown honeys tend to have a higher phenol content compared to light‐yellow honeys, indicating a positive correlation between color intensity and phenol content (Daci et al. 2017). The absorbance in the visible region is attributed to these compounds.

**FIGURE 3:**
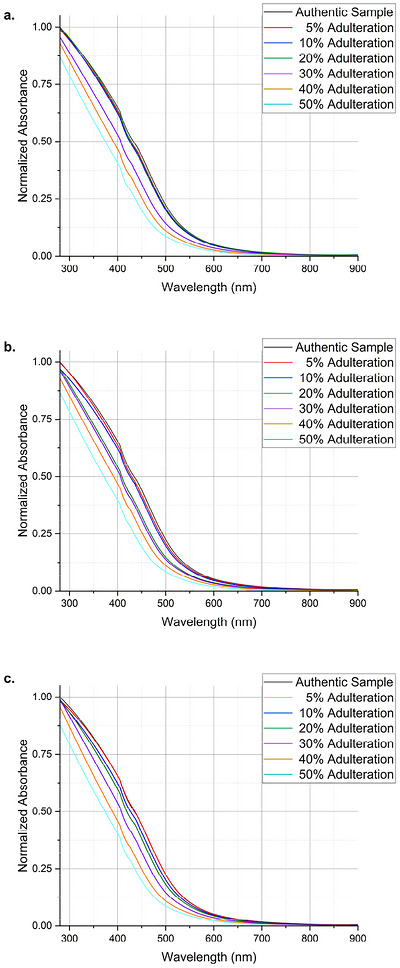
UV‐Vis spectra of the adulterated sample A04 (chestnut) samples with (a) glucose, (b) invert sugar, and (c) maltose syrups.

UV‐Vis spectra demonstrating the representative dilution effects on a single authentic chestnut honeydew honey (Sample A04) are shown in Fig. 3 with (a) glucose, (b) invert sugar, and (c) maltose syrups. Because the spectral baseline shifts and dilution trends were highly consistent across all authentic honey matrices tested, sample A04 is presented as a representative model for the entire dataset. Since adulteration syrups do not contain any coloring compounds, as the adulteration ratio increases, absorbance in the 300–500 nm wavelength region decreases.

### 3.4 Fluorescence Spectroscopy

The fluorescence spectral profile of honey arises from key fluorophores, including phenolic acids, flavonoids, amino acids, and vitamins (Ali et al. 2022). Peaks between 340 and 350 nm are linked to the amino acid tryptophan. These peaks show variations in intensity and slight positional shifts, which may be attributed to changes in protein structure or concentration (Ali et al. 2020). A broad band from 395 to 459 nm in commercial honey samples is associated with fluorescence from Maillard reaction products, which can form during thermal processing. The Maillard reaction, involving the condensation of sugars and amino acids, produces a variety of compounds that may be either toxic/mutagenic or antioxidative (Martins et al. 2000). The emission, spanning from 360 to 420 nm when excited between 250 and 280 nm, is reported to be generated by various phenolic compounds (Karoui et al. 2007).

**FIGURE 4:**
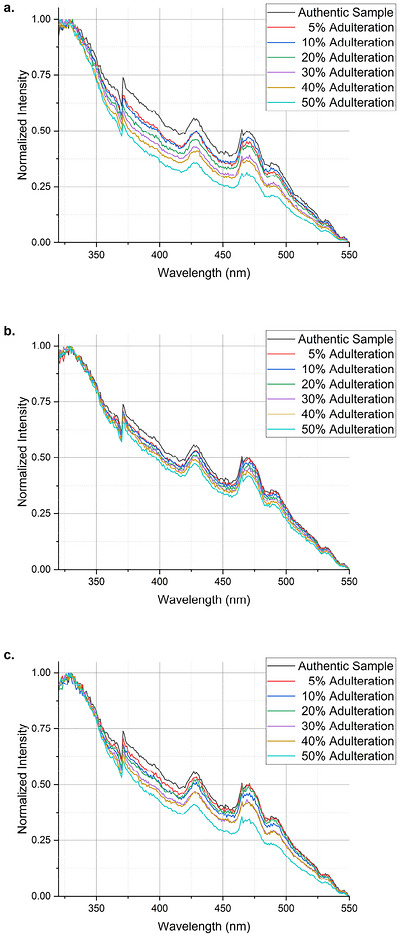
Fluorescence spectra of the adulterated sample A04 (chestnut) samples with (a) glucose, (b) invert sugar, and (c) maltose syrups.

Fluorescence spectra of an example set of adulterated (a) glucose, (b) invert sugar, and (c) maltose syrups vs. a representative authentic chestnut honeydew honey (Sample A04) are shown in Fig. 4. Similar to the pattern recognized in UV‐Vis spectra, the emission intensities and overall baseline decreased consistently across all authentic bases as the adulteration ratio increased; thus, sample A04 effectively represents the comprehensive dataset.

### 3.5 Statistical Analysis

A set of honey samples was randomly selected and adulterated separately with glucose syrup, invert sugar syrup, and maltose syrup. The adulterated samples, consisting of binary mixtures of honey and syrups, were analyzed using PLS‐DA, OPLS‐DA, LightGBM, and XGBoost machine learning models to distinguish authentic honeys from the adulterated ones. The entire spectral range collected from all spectroscopic methods was employed in chemometric analyses. Both raw and transformed data, as described in Section 2.2.6, were utilized to develop binary classification models. The statistical performance parameters, including specificity, sensitivity, and correct classification rates for calibration and external validation, were evaluated. The models that consistently provided accurate results were designated as the final models and reported in Table 3 and Table 4.

**TABLE 3:** Statistical performance metrics of linear latent‐variable models developed separately from rheological and spectroscopic data.

Source	Data	Transformation	Technique	Specificity	Sensitivity	CCR_C_	CCR_V_
**Spectroscopy**	Fluorescence	FD‐SNV	PLS‐DA	0.89 ± 0.19^a^	0.94 ± 0.06^a^	99.33 ± 1.15^ab^	93.33 ± 6.11^a^
	UV‐visible	SD	PLS‐DA	1.00 ± 0.00^a^	0.87 ± 0.00^a^	100.00 ± 0.00^a^	88.00 ± 0.00^a^
	Fluorescence	FD‐SNV	OPLS‐DA	0.89 ± 0.19^a^	0.94 ± 0.06^a^	97.33 ± 4.62^ab^	93.33 ± 6.11^a^
	UV‐visible	FD	OPLS‐DA	1.00 ± 0.00^a^	0.87 ± 0.00^a^	100.00 ± 0.00^a^	88.00 ± 0.00^a^
**Rheology**	Storage mod.	MSC	PLS‐DA	0.62 ± 0.18^a^	0.89 ± 0.06^a^	84.67 ± 3.06^cd^	86.00 ± 5.20^a^
	Loss mod.	SNV	PLS‐DA	1.00 ± 0.00^a^	0.87 ± 0.04^a^	82.67 ± 3.06^d^	87.67 ± 4.51^a^
	Storage mod.	FD‐MSC	OPLS‐DA	0.67 ± 0.29^a^	0.90 ± 0.04^a^	92.00 ± 2.00^bc^	84.67 ± 2.89^a^
	Loss mod.	SNV	OPLS‐DA	0.92 ± 0.14^a^	0.91 ± 0.04^a^	88.67 ± 3.06^cd^	90.67 ± 2.31^a^

UV‐Visible: Ultraviolet visible spectroscopy, FD‐SNV: first derivative standard normal variate, SD: second derivative, PLS‐DA: partial least squares discriminant analysis, OPLS‐DA: orthogonal partial least squares discriminant analysis, MSC: multiplicative scatter correction, SNV: standard normal variate, FD‐MSC: first derivative multiplicative scatter correction, CCR_C_: Correct classification rate for calibration, CCR_V_: Correct classification rate for external validation.

**TABLE 4:** Statistical measures of non‐linear tree‐based gradient boosting models developed separately from rheological and spectral data.

Source	Data	Transformation	Technique	Specificity	Sensitivity	CCR_C_	CCR_V_
Spectroscopy	Fluorescence	FD‐MSC	LightGBM	0.42 ± 0.38^ab^	0.96 ± 0.04^a^	100.00 ± 0.00^a^	88.33 ± 4.04^a^
	UV‐visible	SG	LightGBM	0.33 ± 0.14^ab^	0.96 ± 0.04^a^	98.00 ± 0.00^ab^	87.00 ± 1.73^a^
	Fluorescence	MSC	XGBoost	0.25 ± 0.25^ab^	0.96 ± 0.07^a^	95.33 ± 2.52^bc^	85.67 ± 3.51^a^
	UV‐visible	MSC	XGBoost	0.17 ± 0.14^b^	0.96 ± 0.04^a^	96.67 ± 1.53^abc^	84.67 ± 5.13^a^
Rheology	Storage mod.	FD‐SNV	LightGBM	0.50 ± 0.25^ab^	0.96 ± 0.04^a^	97.33 ± 0.58^ab^	89.33 ± 3.51^a^
	Loss mod.	SD‐SNV	LightGBM	0.75 ± 0.00^a^	0.96 ± 0.07^a^	97.67 ± 0.58^ab^	92.67 ± 5.77^a^
	Storage mod.	SG	XGBoost	0.58 ± 0.14^ab^	0.89 ± 0.02^a^	93.00 ± 2.00^c^	84.67 ± 2.31^a^
	Loss mod.	TD	XGBoost	0.50 ± 0.00^ab^	1.00 ± 0.00^a^	98.67 ± 1.16^ab^	93.00 ± 0.00^a^

UV‐Visible: Ultraviolet visible spectroscopy, FD‐MSC: first derivative multiplicative scatter correction, SG: Savitzky‐Golay, TD: third derivative, LightGBM: light gradient boosting method, XGBoost: eXtreme gradient boosting method, CCR_C_: Correct classification rate for calibration, CCR_V_: Correct classification rate for external validation.

The PLS‐DA model developed using fluorescence spectroscopy data preprocessed with FD‐SNV exhibited strong classification performance, with a specificity of 0.89 ± 0.19 and a sensitivity of 0.94 ± 0.06. The model achieved a very high correct classification rate in calibration (99.33% ± 1.15%) and maintained high performance in external validation (93.33% ± 6.11%).

The PLS‐DA model built on UV‐Vis spectroscopy data using the SD transformation achieved perfect specificity (1.00 ± 0.00), indicating that the positive class was consistently detected across runs. However, sensitivity was comparatively lower and stable (0.87 ± 0.00). This model produced perfect calibration classification (100.00% ± 0.00), while external validation performance decreased to 88.00% ± 0.00%.

Similarly, the OPLS‐DA model constructed from fluorescence spectroscopy data with FD‐SNV preprocessing demonstrated high discriminatory capacity, yielding a specificity of 0.89 ± 0.19 and a sensitivity of 0.94 ± 0.06. The calibration performance was high (97.33% ± 4.62%), and the external validation accuracy remained strong (93.33% ± 6.11%), comparable to the fluorescence PLS‐DA model.

The OPLS‐DA model trained with UV‐Vis spectra after FD transformation again achieved perfect specificity (1.00 ± 0.00) but moderate sensitivity (0.87 ± 0.00). As with the UV‐Vis PLS‐DA counterpart, this model reached perfect calibration performance (100.00% ± 0.00%) yet showed a lower external validation classification rate (88.00% ± 0.00%).

For rheological measurements, the PLS‐DA model based on storage modulus data preprocessed with MSC showed moderate specificity (0.62 ± 0.18) and relatively high sensitivity (0.89 ± 0.06). In contrast to the spectroscopic models, this model exhibited substantially lower calibration performance (84.67% ± 3.06%), while external validation performance was comparable (86.00% ± 5.20%), suggesting limited fit to the training data and modest generalization.

The PLS‐DA model developed using loss modulus data with SNV preprocessing achieved perfect specificity (1.00 ± 0.00), but sensitivity remained at 0.87 ± 0.04. Although calibration accuracy was comparatively low (82.67% ± 3.06%), validation performance improved (87.67% ± 4.51%).

The OPLS‐DA model using storage modulus data after FD‐MSC transformation produced slightly higher specificity (0.67 ± 0.29) and sensitivity (0.90 ± 0.04) than the corresponding storage‐modulus PLS‐DA model. This model achieved a notably improved calibration classification rate (92.00% ± 2.00%) but a lower external validation rate (84.67% ± 2.89%).

Finally, the OPLS‐DA model trained on loss modulus data with SNV transformation displayed the most balanced performance among the rheological models, with high specificity (0.92 ± 0.14) and sensitivity (0.91 ± 0.04). This balance was accompanied by improved calibration (88.67% ± 3.06%) and strong external validation performance (90.67% ± 2.31%), suggesting a more stable discrimination pattern relative to the other rheology‐based models.

Across the evaluated models, spectroscopic approaches generally yielded numerically higher external validation correct classification rates, particularly for fluorescence‐based models (93.33% ± 6.11%), coupled with consistently high sensitivity (0.94 ± 0.06). Yet all models belonged to the same significance group, indicating statistically comparable validation performance (p > 0.05). UV‐Vis models achieved perfect specificity (1.00 ± 0.00) in both PLS‐DA and OPLS‐DA, but this came with reduced sensitivity (0.87 ± 0.00) and a clear drop in external validation performance (88.00% ± 0.00%), indicating a more pronounced trade‐off between detecting the authentic samples and avoiding false positives. In rheological data, performance depended strongly on the modulus type and preprocessing: storage modulus models showed moderate specificity (0.62–0.67) and mid‐to‐high sensitivity (0.89–0.90), while loss modulus models achieved higher specificity (0.92–1.00) (all within the same significance group) with similar sensitivity (0.87–0.91). Notably, the loss modulus OPLS‐DA model with SNV preprocessing provided the best balance between specificity and sensitivity and yielded the strongest external validation performance within the rheological set (90.67% ± 2.31%), supporting loss modulus as a comparatively robust rheological feature for classification when paired with appropriate preprocessing and model structure.

The model developed using fluorescence spectroscopy data transformed with FD+MSC and analyzed using the LightGBM algorithm exhibited good calibration performance (100.0% ± 0.00%) and high validation accuracy (88.33% ± 4.04%). While the sensitivity was consistently high (0.96 ± 0.04), the specificity was relatively low (0.42 ± 0.38) and highly variable.

The LightGBM model developed with UV‐Visible spectral data preprocessed using SG transformation demonstrated similarly high sensitivity (0.96 ± 0.04), with correct classification rates of 98.00% for calibration and 87.00% for validation. However, the specificity remained low (0.33 ± 0.14).

The XGBoost model constructed using fluorescence spectroscopy data with MSC transformation yielded calibration and validation accuracies of 95.33% ± 2.52% and 85.67% ± 3.51%, respectively. The sensitivity remained high (0.960 ± 0.069), while specificity dropped to 0.25 ± 0.25.

The UV‐Visible spectral data analyzed with XGBoost after MSC transformation showed slightly higher calibration performance (96.67% ± 1.52%) and comparable validation accuracy (84.67% ± 5.13%) relative to the fluorescence counterpart. However, the specificity further declined to 0.166 ± 0.144, while the model was reliable in detecting adulterated samples (sensitivity = 0.96 ± 0.04).

In contrast, the LightGBM model trained with storage modulus data preprocessed using FD+SNV transformation displayed balanced and promising performance. With specificity at 0.50 ± 0.25 and sensitivity at 0.96 ± 0.04, the model achieved calibration and validation accuracies of 97.33% ± 0.58% and 89.33% ± 3.51%, respectively.

The LightGBM model utilizing loss modulus data with SD+SNV transformation yielded the highest specificity among all models (0.75 ± 0.00), along with high sensitivity (0.96 ± 0.07), calibration accuracy (97.67% ± 0.58%), and validation accuracy (92.67% ± 5.77%).

The XGBoost model based on storage modulus data preprocessed with SG transformation showed slightly lower performance, with calibration and validation accuracies of 93.00% ± 2.00% and 84.67% ± 2.31%, respectively. Specificity was moderate (0.58 ± 0.14), and sensitivity dropped slightly to 0.89 ± 0.02.

Finally, the XGBoost model trained on loss modulus data transformed with TD achieved high sensitivity (1.00 ± 0.00) and high calibration (98.67% ± 1.16%) and validation (93.00% ± 0.00%) accuracy values; the only limitation was a moderate specificity of 0.50 ± 0.00.

In the fluorescence profiles, the emission intensities and overall baseline levels exhibited a continuous decrease across all authentic bases as a function of the increasing adulteration ratio. This spectral behavior highlights a qualitative reduction in natural fluorophores due to the incorporation of the uncolored syrup, providing a reliable physical basis for the chemometric classification.

In alignment with these fluorescence trends, a progressive decline in absorbance within the 300–500 nm wavelength range was observed in the UV‐Vis spectra with increasing levels of adulteration. This parallel behavior is closely linked to the physicochemical nature of the adulterant; since the formulation syrups lack inherent coloring compounds, they systematically dilute the chromophoric density of the authentic matrix.

The loss modulus models achieved a more balanced classification performance compared to those based on the storage modulus, largely due to the viscous nature of the honey matrix. Since authentic honey is predominantly a viscous liquid, its storage modulus values are extremely low at lower temperatures, which can introduce numerical noise or fall near the sensitivity limits of the rheometer. While storage modulus is effective at detecting the sharp, threshold‐driven onset of solid‐like properties caused by the gelatinization of starch‐based polymers in the adulterants, it lacks a consistent baseline in pure samples. In contrast, loss modulus directly reflects the viscous energy dissipation and molecular friction within the carbohydrate‐water matrix across the full 20°C–80°C range. Because adulteration alters the hydrogen‐bonding networks and flow dynamics continuously as temperature rises, loss modulus provides a more stable and numerically consistent dataset for tree‐based algorithms. This stability allows the models to better distinguish natural botanical variations from the structural changes induced by adulteration.

Across the evaluated machine‐learning models, spectroscopic inputs (fluorescence and UV‐Vis) consistently produced strong and statistically equivalent overall correct classification rates in both calibration (95.33%–100.00%) and external validation (84.67%–88.33%) and maintained uniformly high sensitivity (≈0.96), indicating reliable identification of adulterated samples (negative class). However, these high accuracies were accompanied by persistently low and often highly variable specificity (0.17–0.42), demonstrating a systematic limitation in correctly recognizing authentic samples (positive class) and suggesting that model decisions were biased toward the negative class despite favorable CCR metrics. Although LightGBM numerically outperformed XGBoost in validation accuracy for spectral data, Tukey's post‐hoc test confirmed that neither algorithm statistically improved specificity within the spectroscopic set, as they largely shared the same significance groups.

In contrast, rheological features yielded more balanced class‐wise performance, with several models improving specificity while preserving high sensitivity. The LightGBM model using storage modulus data with FD+SNV preprocessing provided modest but more even discrimination (specificity = 0.50 ± 0.25; sensitivity = 0.96 ± 0.04) and achieved solid validation accuracy (89.33% ± 3.51). Most notably, the LightGBM model built on loss modulus data with SD+SNV preprocessing delivered the best overall balance across all models, combining the highest specificity (0.75 ± 0.00) with consistently high sensitivity (0.96 ± 0.07) and strong validation performance (92.67% ± 5.77), highlighting loss modulus as a particularly robust rheological descriptor for authentic‐adulterated discrimination when paired with appropriate preprocessing. Among the XGBoost rheology models, the loss modulus model with TD transformation achieved excellent sensitivity (1.00 ± 0.00) and the highest validation accuracy (93.00% ± 0.00), but its only remaining constraint was moderate specificity (0.50 ± 0.00), indicating that improvements in overall accuracy did not necessarily translate into better detection of authentic samples. Overall, these findings indicate that rheological data (especially loss modulus) offer superior discriminatory power in terms of balanced specificity‐sensitivity trade‐offs, whereas spectral data, while accurate in aggregate, tend to under‐detect the authentic class under the current modeling and preprocessing configurations.

Comparing the two modeling frameworks, clear and complementary performance profiles emerged. The chemometric discriminant models (PLS‐DA/OPLS‐DA) generally delivered a more favorable class‐wise balance for the authentic (negative) class, with markedly higher specificity values—particularly in spectroscopy, where fluorescence models reached 0.89 ± 0.19 specificity with high sensitivity (0.94 ± 0.06), and UV‐Vis models achieved perfect specificity (1.00 ± 0.00), albeit at the cost of reduced sensitivity (0.87 ± 0.00) and lower external validation CCR (88.00% ± 0.00). In contrast, the machine‐learning models (LightGBM/XGBoost) tended to prioritize discrimination of the adulterated (positive) class, exhibiting consistently high sensitivity across both spectral and rheological inputs (typically 0.96–1.00) and strong overall CCRs, but with persistently low—and in some cases highly variable‐specificity for authentic samples when spectral data were used (0.17–0.42). This indicates a systematic tendency of the tree‐based classifiers to under‐detect the authentic class despite favorable aggregate accuracy metrics. Notably, this gap narrowed when rheological features were introduced: loss modulus models, particularly LightGBM with SD+SNV preprocessing, achieved substantially improved specificity (0.75 ± 0.00) while maintaining high sensitivity (0.96 ± 0.07) and strong validation performance (92.67% ± 5.77), approaching or surpassing the practical balance observed in the best PLS‐DA/OPLS‐DA configurations. Overall, PLS‐DA/OPLS‐DA provided stronger authentic‐class capture in spectroscopy, whereas LightGBM/XGBoost required rheological descriptors—especially loss modulus—to achieve comparably balanced discrimination without sacrificing sensitivity.

## 4 Conclusion

This study independently evaluated thermorheological and spectroscopic analyses for detecting honey adulteration, applying oscillatory temperature‐sweep tests, UV‐Vis spectroscopy, and fluorescence spectroscopy to samples adulterated with glucose, invert sugar, and maltose syrups at 5%–50% addition levels. Linear latent‐variable classifiers (PLS‐DA and OPLS‐DA) and non‐linear gradient boosting models (LightGBM and XGBoost) were systematically compared for spectral and rheological data across multiple pre‐processing schemes using repeated calibration and independent validation splits. Spectroscopy‐based latent‐variable models provided strong discrimination, particularly fluorescence (external validation CCR ≈ 93%, specificity ≈ 0.89, and sensitivity ≈ 0.94), whereas UV‐Vis achieved perfect specificity (1.00) but at lower sensitivity (≈0.87). Tree‐based models applied to spectral data maintained high sensitivity (≈0.96) and good CCR (≈85‐88%) but exhibited low specificity (0.17‐0.42), indicating under‐recognition of authentic samples. In contrast, rheological descriptors, especially the loss modulus, achieved a more balanced performance: the best configuration (loss modulus + SD‐SNV + LightGBM) reached 0.75 specificity, ≈0.96 sensitivity, and ≈93% external validation CCR, while XGBoost achieved similarly high CCR with perfect sensitivity in one setting. Serving as a proof‐of‐concept, these results demonstrate that thermorheology, analyzed independently with both linear and non‐linear modeling approaches, provides a robust tool for qualitative (but not quantitative) honey adulteration detection. Each analytical approach was evaluated independently, ensuring that performance metrics reflect the separate discriminative power of thermorheological and spectroscopic data. However, the relatively small sample size and use of adulterated samples derived from a limited number of authentic parent concentrates utilized in this study represent a primary limitation.

To ensure the robustness required for practical industrial applications and routine monitoring, the findings of this study should be interpreted with consideration of the limited authentic dataset and the possibility of parental leakage. Future research should expand upon these initial findings by incorporating larger and more diverse datasets. Additionally, addressing the mild imbalance between authentic and adulterated classes through more extensive sampling across a broader range of botanical and geographical origins will be essential to further enhance model specificity and generalize the findings for large‐scale quality control.

## Author Contributions

**Bilge Basturk Berk**: conceptualization, methodology, investigation, data curation, writing – original draft. **Berkay Berk**: conceptualization, methodology, investigation, data curation, writing – original draft. **Cagri Cavdaroglu**: conceptualization, methodology, investigation, data curation, writing – original draft. **Neslihan Bozdogan**: conceptualization, methodology, data curation, investigation, writing – original draft. **Sebnem Tavman**: conceptualization, supervision, writing – review and editing. **Seher Kumcuoglu**: conceptualization, supervision, writing – review and editing. **Sevcan Unluturk**: conceptualization, supervision, writing – review and editing.

## Ethics Statement

Ethics approval was not required for this research.

## Conflicts of Interest

The authors declare no conflicts of interest.

## Supporting information




**Supplementary Material**: jfds71228‐sup‐0001‐SuppMat.docx

**Supplementary Material**: jfds71228‐sup‐0002‐SuppMat.docx

## Data Availability

The data that support the findings of this study are available from the corresponding author upon reasonable request.

## References

[jfds71228-bib-0001] Ali, H. , S. Khan , R. Ullah , and B. Khan . 2020. “Fluorescence Fingerprints of Sidr Honey in Comparison With Uni/Polyfloral Honey Samples.” European Food Research and Technology 246, no. 9: 1829–1837. 10.1007/s00217-020-03536-6.

[jfds71228-bib-0002] Ali, H. , K. Rafique , R. Ullah , M. Saleem , and I. Ahmad . 2022. “Classification of Sidr Honey and Detection of Sugar Adulteration Using Right Angle Fluorescence Spectroscopy and Chemometrics.” European Food Research and Technology 248, no. 7: 1823–1829. 10.1007/s00217-022-04008-9. 35431646 PMC8994421

[jfds71228-bib-0003] Amaral‐Fonseca, M. , R. Morellon‐Sterling , R. Fernández‐Lafuente , and P. W. Tardioli . 2020. “Optimization of Simultaneous Saccharification and Isomerization of Dextrin to High Fructose Syrup Using a Mixture of Immobilized Amyloglucosidase and Glucose Isomerase.” Catalysis Today 362: 40–47. 10.1016/j.cattod.2020.03.021.

[jfds71228-bib-0004] Amiry, S. , M. Esmaiili , and M. Alizadeh . 2017. “Classification of Adulterated Honeys by Multivariate Analysis.” Food Chemistry 224: 390–397. 10.1016/j.foodchem.2016.12.025. 28159285

[jfds71228-bib-0005] Becerril‐Sánchez, A. L. , B. Quintero‐Salazar , O. Dublán‐García , and H. B. Escalona‐Buendía . 2021. “Phenolic Compounds in Honey and Their Relationship With Antioxidant Activity, Botanical Origin, and Color.” Antioxidants 10: 1700. 10.3390/antiox10111700. 34829570 PMC8614671

[jfds71228-bib-0006] Berk, B. , C. Cavdaroglu , L. Grunin , et al. 2022. “Use of Magic Sandwich Echo and Fast Field Cycling NMR Relaxometry on Honey Adulteration With Corn Syrup.” Journal of the Science of Food and Agriculture 102, no. 7: 2667–2675. 10.1002/jsfa.11606. 34713450

[jfds71228-bib-0007] Bertelli, D. , M. Lolli , G. Papotti , L. Bortolotti , G. Serra , and M. Plessi . 2010. “Detection of Honey Adulteration by Sugar Syrups Using One‐Dimensional and Two‐Dimensional High‐Resolution Nuclear Magnetic Resonance.” Journal of Agricultural and Food Chemistry 58, no. 15: 8495–8501. 10.1021/jf101460t. 20681637

[jfds71228-bib-0008] Cardona, Y. , A. Torres , W. Hoffmann , and I. Lamprecht . 2018. “Differentiation of Honey From Melipona Species Using Differential Scanning Calorimetry.” Food Analytical Methods 11, no. 4: 1056–1067. 10.1007/s12161-017-1083-z.

[jfds71228-bib-0009] Cavdaroglu, C. , and B. Ozen . 2022. “Detection of Vinegar Adulteration With Spirit Vinegar and Acetic Acid Using UV–Visible and Fourier Transform Infrared Spectroscopy.” Food Chemistry 379: 132150. 10.1016/J.FOODCHEM.2022.132150. 35065489

[jfds71228-bib-0010] Chen, Q. , S. Qi , H. Li , X. Han , Q. Ouyang , and J. Zhao . 2014. “Determination of Rice Syrup Adulterant Concentration in Honey Using Three‐Dimensional Fluorescence Spectra and Multivariate Calibrations.” Spectrochimica Acta Part A: Molecular and Biomolecular Spectroscopy 131: 177–182. 10.1016/j.saa.2014.04.071. 24830631

[jfds71228-bib-0011] Chen, T. , T. He , M. Benesty , et al. 2022. xgboost: Extreme Gradient Boosting . https://cran.r‐project.org/package=xgboost.

[jfds71228-bib-0013] Çinar, S. B. , A. Ekşi , and İ. Coşkun . 2014. “Carbon Isotope Ratio (13C/12C) of Pine Honey and Detection of HFCS Adulteration.” Food Chemistry 157: 10–13. 10.1016/j.foodchem.2014.02.006. 24679745

[jfds71228-bib-0014] Codex Alimentarius Commission . 2001. Codex Alimentarius Commission Standards. Codex Standard for Honey 12–1981.

[jfds71228-bib-0015] Daci, M. , A. Mehmeti , L. Zeneli , and N. Daci . 2017. “Evaluation of Antioxidant Activity, Heavy Metals and Colour Intensity of Honeys From Different Parts of Kosovo.” Journal of Environmental Protection and Ecology 18: 737–748.

[jfds71228-bib-0016] Dimakopoulou‐Papazoglou, D. , N. Ploskas , S. Serrano , et al. 2023. “Application of UV–Vis Spectroscopy for the Detection of Adulteration in Mediterranean Honeys.” European Food Research and Technology 249, no. 12: 3043–3053. 10.1007/s00217-023-04347-1.

[jfds71228-bib-0017] Everstine, K. , J. Spink , and S. Kennedy . 2013. “Economically Motivated Adulteration (EMA) of Food: Common Characteristics of EMA Incidents.” Journal of Food Protection 76, no. 4: 723–735. 10.4315/0362-028X.JFP-12-399. 23575142

[jfds71228-bib-0018] Fakhlaei, R. , A. A. Babadi , N. M. Ariffin , and Z. Xiaobo . 2025. “Development of FTIR‐ATR Spectra and PLS Regression Combination Model for Discrimination of Pure and Adulterated Acacia Honey.” Food Control 169: 110996. 10.1016/j.foodcont.2024.110996.

[jfds71228-bib-0019] Huang, F. , H. Song , L. Guo , et al. 2020. “Detection of Adulteration in Chinese Honey Using NIR and ATR‐FTIR Spectral Data Fusion.” Spectrochimica Acta Part A: Molecular and Biomolecular Spectroscopy 235: 118297. 10.1016/j.saa.2020.118297. 32248033

[jfds71228-bib-0020] Karoui, R. , E. Dufour , J.‐O. Bosset , and J. De Baerdemaeker . 2007. “The Use of Front Face Fluorescence Spectroscopy to Classify the Botanical Origin of Honey Samples Produced in Switzerland.” Food Chemistry 101, no. 1: 314–323. 10.1016/j.foodchem.2006.01.039.

[jfds71228-bib-0021] Li, C. , X. Fu , F. Luo , and Q. Huang . 2013. “Effects of Maltose on Stability and Rheological Properties of Orange Oil‐in‐Water Emulsion Formed by OSA Modified Starch.” Food Hydrocolloids 32, no. 1: 79–86. 10.1016/j.foodhyd.2012.12.004.

[jfds71228-bib-0022] Martins, S. I. F. S. , W. M. F. Jongen , and M. A. J. S. van Boekel . 2000. “A Review of Maillard Reaction in Food and Implications to Kinetic Modelling.” Trends in Food Science & Technology 11, no. 9: 364–373. 10.1016/S0924-2244(01)00022-X.

[jfds71228-bib-0023] Miśkiewicz, K. , J. Rosicka‐Kaczmarek , and E. Nebesny . 2020. “Effects of Chickpea Protein on Carbohydrate Reactivity in Acrylamide Formation in Low Humidity Model Systems.” Foods 9, no. 2: 167. 10.3390/foods9020167. 32050683 PMC7073537

[jfds71228-bib-0024] Momtaz, M. , S. Y. Bubli , and M. S. Khan . 2023. “Mechanisms and Health Aspects of Food Adulteration: A Comprehensive Review.” Foods 12, no. 1: 199. 10.3390/foods12010199. 36613416 PMC9818512

[jfds71228-bib-0025] Oroian, M. , S. Ropciuc , S. Paduret , and E. Todosi . 2018. “Rheological Analysis of Honeydew Honey Adulterated With Glucose, Fructose, Inverted Sugar, Hydrolysed Inulin Syrup and Malt Wort.” LWT 95: 1–8. 10.1016/j.lwt.2018.04.064.

[jfds71228-bib-0026] Ruiz‐Matute, A. I. , S. Rodríguez‐Sánchez , M. L. Sanz , and I. Martínez‐Castro . 2010. “Detection of Adulterations of Honey With High Fructose Syrups From Inulin by GC Analysis.” Journal of Food Composition and Analysis 23, no. 3: 273–276. 10.1016/j.jfca.2009.10.004.

[jfds71228-bib-0027] Särndal, C.‐E. , B. Swensson , and J. Wretman . 1992. Model Assisted Survey Sampling.1st ed. Springer. https://link.springer.com/book/9780387406206.

[jfds71228-bib-0028] Shi, Y. , G. Ke , D. Soukhavong , et al. 2023. lightgbm: Light Gradient Boosting Machine . https://cran.r‐project.org/package=lightgbm.

[jfds71228-bib-0029] Thévenot, E. A. , A. Roux , Y. Xu , E. Ezan , and C. Junot . 2015. “Analysis of the Human Adult Urinary Metabolome Variations With Age, Body Mass Index, and Gender by Implementing a Comprehensive Workflow for Univariate and OPLS Statistical Analyses.” Journal of Proteome Research 14, no. 8: 3322–3335. 10.1021/acs.jproteome.5b00354. 26088811

[jfds71228-bib-0030] Walther, M. , B. M. Fischer , and P. Uhd Jepsen . 2003. “Noncovalent Intermolecular Forces in Polycrystalline and Amorphous Saccharides in the Far Infrared.” Chemical Physics 288, no. 2: 261–268. 10.1016/S0301-0104(03)00031-4.

[jfds71228-bib-0031] Xue, X. , Q. Wang , Y. Li , et al. 2013. “2‐Acetylfuran‐3‐Glucopyranoside as a Novel Marker for the Detection of Honey Adulterated With Rice Syrup.” Journal of Agricultural and Food Chemistry 61, no. 31: 7488–7493. 10.1021/jf401912u. 23844945

[jfds71228-bib-0032] Yan, S. , M. Sun , X. Wang , J. Shan , and X. Xue . 2022. “A Novel, Rapid Screening Technique for Sugar Syrup Adulteration in Honey Using Fluorescence Spectroscopy.” Foods 11, no. 15: 2316. 10.3390/foods11152316. 35954081 PMC9368237

[jfds71228-bib-0033] Yeole, A. N. , G. P. M S , and S. Kumar . 2024. “Robust Honey Adulteration Detection through Ensemble Techniques With Hyperspectral Analysis.” 2024 International Conference on Artificial Intelligence and Emerging Technology (Global AI Summit) , 298–305. IEEE. 10.1109/GlobalAISummit62156.2024.10947943.

[jfds71228-bib-0034] Yilmaz, M. T. , N. B. Tatlisu , O. S. Toker , et al. 2014. “Steady, Dynamic and Creep Rheological Analysis as a Novel Approach to Detect Honey Adulteration by Fructose and Saccharose Syrups: Correlations With HPLC‐RID Results.” Food Research International 64: 634–646. 10.1016/j.foodres.2014.07.009. 30011698

