# Supplementary material for: Detection of Honey Adulteration Using Thermorheological and Spectroscopic Analyses: Independent Evaluation With Linear Latent‐Variable and Gradient Boosting Models
Source: J Food Sci. 2026 Jun 26;91(7):e71228. doi: 10.1111/1750-3841.71228 (PMC13308385; doi:10.1111/1750-3841.71228)
Supplement: Supplementary file 1 — Supplementary Material: jfds71228‐sup‐0001‐SuppMat.docx [file JFDS-91-0-s001.docx]

**Supplementary File A**

**Storage and loss moduli thermorheograms of all samples**

Fig. S1. A01 honey adulteration with S1 syrup

Fig. S2. A01 honey adulteration with S2 syrup

Fig. S3. A01 honey adulteration with S3 syrup

Fig. S4. A02 honey adulteration with S1 syrup

Fig. S5. A02 honey adulteration with S2 syrup

Fig. S6. A02 honey adulteration with S3 syrup

Fig. S7. A03 honey adulteration with S1 syrup

Fig. S8. A03 honey adulteration with S2 syrup

Fig. S9. A03 honey adulteration with S3 syrup

Fig. S10. A04 honey adulteration with S1 syrup

Fig. S11. A04 honey adulteration with S2 syrup

Fig. S12. A04 honey adulteration with S3 syrup

**Tanδ thermorheograms of all samples**

Fig. S12. A04 honey adulteration with S1 syrup

Fig. S12. A04 honey adulteration with S2 syrup

Fig. S12. A04 honey adulteration with S3 syrup

Fig. S12. A03 honey adulteration with S1 syrup

Fig. S12. A03 honey adulteration with S2 syrup

Fig. S12. A03 honey adulteration with S3 syrup

Fig. S12. A02 honey adulteration with S1 syrup

Fig. S12. A02 honey adulteration with S2 syrup

Fig. S12. A02 honey adulteration with S3 syrup

Fig. S12. A01 honey adulteration with S1 syrup

Fig. S12. A01 honey adulteration with S2 syrup

Fig. S12. A01 honey adulteration with S3 syrup
