# Supplementary material for: Detection of Honey Adulteration Using Thermorheological and Spectroscopic Analyses: Independent Evaluation With Linear Latent‐Variable and Gradient Boosting Models
Source: J Food Sci. 2026 Jun 26;91(7):e71228. doi: 10.1111/1750-3841.71228 (PMC13308385; doi:10.1111/1750-3841.71228)
Supplement: Supplementary file 2 — Supplementary Material: jfds71228‐sup‐0002‐SuppMat.docx [file JFDS-91-0-s002.docx]

Supplementary File B

Table S1: Crossover points of the samples

| **Sample** | **Crossover Point 1 (°C)** | **Crossover Point 2 (°C)** |
| --- | --- | --- |
| **A01** | − | − |
| **A01_S1_5** | − | − |
| **A01_S1_10** | − | − |
| **A01_S1_20** | − | − |
| **A01_S1_30** | 38.13 | 78.50 |
| **A01_S1_40** | 34.83 | − |
| **A01_S1_50** | 33.98 | − |
| **A01_S2_5** | − | − |
| **A01_S2_10** | − | − |
| **A01_S2_20** | − | − |
| **A01_S2_30** | − | − |
| **A01_S2_40** | − | − |
| **A01_S2_50** | − | − |
| **A01_S3_5** | − | − |
| **A01_S3_10** | − | − |
| **A01_S3_20** | − | − |
| **A01_S3_30** | 40.31 | 77.88 |
| **A01_S3_40** | 35.67 | − |
| **A01_S3_50** | 36.44 | − |
| **A02** | − | − |
| **A02_S1_5** | 26.85 | 67.00 |
| **A02_S1_10** | 33.90 | 68.91 |
| **A02_S1_20** | 31.63 | 74.30 |
| **A02_S1_30** | 29.10 | − |
| **A02_S1_40** | 28.34 | − |
| **A02_S1_50** | 26.77 | − |
| **A02_S2_5** | − | − |
| **A02_S2_10** | − | − |
| **A02_S2_20** | − | − |
| **A02_S2_30** | − | − |
| **A02_S2_40** | − | − |
| **A02_S2_50** | − | − |
| **A02_S3_5** | 43.11 | 51.08 |
| **A02_S3_10** | 37.71 | 66.11 |
| **A02_S3_20** | 33.50 | 68.03 |
| **A02_S3_30** | 32.39 | 77.95 |
| **A02_S3_40** | 31.04 | − |
| **A02_S3_50** | 30.93 | − |
| **A03** | − | − |
| **A03_S1_5** | − | − |
| **A03_S1_10** | 47.49 | 56.71 |
| **A03_S1_20** | 35.29 | 71.44 |
| **A03_S1_30** | 33.42 | 77.64 |
| **A03_S1_40** | 31.34 | − |
| **A03_S1_50** | 29.01 | − |
| **A03_S2_5** | − | − |
| **A03_S2_10** | − | − |
| **A03_S2_20** | − | − |
| **A01_S2_30** | − | − |
| **A03_S2_40** | − | − |
| **A03_S2_50** | − | − |
| **A03_S3_5** | − | − |
| **A03_S3_10** | − | − |
| **A03_S3_20** | − | − |
| **A03_S3_30** | 38.18 | 66.78489 |
| **A03_S3_40** | 34.75 | − |
| **A03_S3_50** | 30.62 | − |
| **A04** | − | − |
| **A04_S1_5** | 38.10 | 56.85 |
| **A04_S1_10** | 34.91 | 65.79 |
| **A04_S1_20** | 28.44 | − |
| **A04_S1_30** | 33.21 | 78.21 |
| **A04_S1_40** | 31.63 | − |
| **A04_S1_50** | 31.26 | − |
| **A04_S2_5** | − | − |
| **A04_S2_10** | − | − |
| **A04_S2_20** | − | − |
| **A04_S2_30** | − | − |
| **A04_S2_40** | − | − |
| **A04_S2_50** | − | − |
| **A04_S3_5** | − | − |
| **A04_S3_10** | − | − |
| **A04_S3_20** | 39.19 | 69.21 |
| **A04_S3_30** | 34.50 | 75.48 |
| **A04_S3_40** | 28.14 | − |
| **A04_S3_50** | 27.83 | − |
